# *SIL-TAL1* Rearrangement is Related with Poor Outcome: A Study from a Chinese Institution

**DOI:** 10.1371/journal.pone.0073865

**Published:** 2013-09-09

**Authors:** Di Wang, Guangrong Zhu, Na Wang, Xiaoxi Zhou, Yunfan Yang, Shiqiu Zhou, Jie Xiong, Jing He, Lijun Jiang, Chunrui Li, Danmei Xu, Liang Huang, Jianfeng Zhou

**Affiliations:** 1 Department of Hematology, Tongji Hospital, Tongji Medical College, Huazhong University of Science and Technology, Wuhan, Hubei, The People's Republic of China; 2 Department of Hematology, Affiliated Hospital of Nanjing University of Traditional Chinese Medicine, Nanjing, Jiangsu, The People's Republic of China; UNIVERSITY MAGNA GRAECIA, Italy

## Abstract

*SIL-TAL1* rearrangement is common in T-cell acute lymphoblastic leukemia (T-ALL), however its prognostic implication remains controversial. To investigate the clinical characteristics and outcome of this subtype in Chinese population, we systemically reviewed 62 patients with newly diagnosed T-ALL, including 15 patients with *SIL-TAL1* rearrangement. We found that *SIL-TAL1^+^* T-ALL was characterized by higher white blood cell count (*P* = 0.029) at diagnosis, predominant cortical T-ALL immunophenotype (*P* = 0.028) of the leukemic blasts, and a higher prevalence of tumor lysis syndrome (TLS, *P*<0.001) and disseminated intravascular coagulation (DIC, *P*<0.001), which led to a higher early mortality (*P* = 0.011). Compared with *SIL-TAL1^−^* patients, *SIL-TAL1^+^* patients had shorter relapse free survival (*P* = 0.007) and overall survival (*P* = 0.002). Our NOD/SCID xenotransplantation model also demonstrated that *SIL-TAL1^+^* mice models had earlier disease onset, higher leukemia cell load in peripheral blood and shorter overall survival (*P*<0.001). Moreover, the *SIL-TAL1^+^* mice models exerted a tendency of TLS/DIC and seemed vulnerable towards chemotherapy, which further simulated our clinical settings. These data demonstrate that *SIL-TAL1* rearrangement identifies a distinct subtype with inferior outcome which could allow for individual therapeutic stratification for T-ALL patients.

## Introduction

T-cell acute lymphoblastic leukemia (T-ALL) accounts for approximately 15% and 25% of the newly diagnosed ALL in children and adults, respectively, and is often linked with a poor prognosis. T-cell acute lymphoblastic leukemia 1 (*TAL1*, also known as stem cell leukemia, *SCL*) is a frequent target for chromosomal translocation, interstitial deletion, or mutation in T-ALL [1], [2]. It encodes a class II basic-helix-loop-helix (bHLH) transcription factor that is a master regulator for hematopoietic lineage commitment. *TAL1* has been proved essential for the development of mouse HSCs [3]. *SIL-TAL1* rearrangement is a common *TAL1* related alteration and occurs in 16∼26% of T-ALL cases. It results from a 90 kb-interstitial-deletion in the *TAL1* gene locus that fuses with the 5` non-coding portion of *SIL*, thus leading to an aberrant overexpression of *TAL1* protein [4], [5].

Patients bearing *SIL-TAL1* rearrangement (*SIL-TAL1^+^*) are defined by distinct clinical and biological characteristics such as a high white-blood-cell count and hemoglobin, T-lineage immunophenotype with CD2 expression, and low incidence in adult patients [6]–[8]. Some of these features are generally associated with more unfavorable clinical features. However, the prognosis of this rearrangement remains controversial [6], [7], [9]–[12]. *TAL1* rearrangements had been historically linked with a better outcome in some reports [7], [10], [12]. With respect to *SIL-TAL1*, the results from Mansur MB indicated a negative impact on overall survival (OS) in Brazilian pediatric patients [6], whereas Ballerini P suggested that *SIL-TAL1* expression did not significantly affect either leukemia-free survival (LFS) or OS [11]. Therefore, the clinical features and prognostic significance of *SIL-TAL1* rearrangement deserves further study.

In the present study, we retrospectively reviewed a serial of 62 patients diagnosed with T-ALL in our department. Clinical characteristics and outcome was compared for patient subgroup with and without *SIL-TAL1* rearrangement. Moreover, we established several reliable T-ALL xenograft murine models, which would further demonstrate the disease phenotype and responses to chemotherapy of this distinct T-ALL subtype.

## Materials and Methods

### Ethics Statement

Informed consent had been signed by each patient at diagnosis. This study was approved by the Research Ethics Committee of Tongji Hospital of Tongji Medical College, Huazhong University of Science and Technology, Wuhan, China. Animal care and experimentation were conducted in accordance with guidelines of the Institutional Committee of Animal Care and Treatment in Tongji Hospital of Tongji Medical College, Huazhong University of Science and Technology, Wuhan, China.

### Clinical Comparison of T-ALL Patients

We systemically collected clinical data from 62 *de novo* T-ALL patients hospitalized in our department from 2006 to 2012. The bone marrow specimen of each patient was obtained before treatment and the diagnosis was made based on a multiparametric approach, including examination of clinical characteristics, morphologic features, immunophenotype, and cytogenetic and molecular findings [13]. *SIL-TAL1* fusion transcript was detected using reverse transcription polymerase chain reaction (RT-PCR) [14]. The PCR primer pairs were: F 5′-TCCCGCTCCTACCCTGCAA-3′ and R 5′-CGTCGCGGCCCTTTAAGTC-3′ [14]. The evaluation criteria for tumor lysis syndrome (TLS) and disseminated intravascular coagulation (DIC) was as described by Tosi *et al* [15] and Taylor *et al* [16], respectively. Clinical characteristics and outcome was compared for patients with and without *SIL-TAL1* rearrangement. Complete remission (CR) was defined as bone marrow morphology with less than 5% blasts, a neutrophil count of 1×10^9^/L or more, a platelet count of 100×10^9^/L or more, and no evidence of extramedullary leukemia. Overall survival (OS) was calculated from the first day of induction therapy to their death or the last day of observation. Relapse free survival (RFS) was calculated from the initiation of induction therapy.

### Comparison of *SIL-TAL1^+^* and *SIL-TAL1^−^* T-ALL in Xenograft Models

We had inoculated leukemia cells from four T-ALL patients, including one *SIL-TAL1^+^* patient, to NOD/SCID mice [17]. Clinical data of the four patients and detailed protocols for xenotransplantation and evaluation had been described previously [17]. In this study, data obtained from the secondary passage (P2) of these murine models were reanalyzed (Table S1). Comparison of disease phenotype and survival was made between the mice with and without *SIL-TAL1* rearrangement. The disease onset was defined as the first time human CD45^+^ cells detected in peripheral blood. Overall survival of the mice was calculated from disease onset till the death.

### *In vivo* Drug Treatment of *SIL-TAL1^+^* Murine Models

In this study, we serial transplanted leukemia cells from the secondary passage of *SIL-TAL1^+^* model to NOD/SCID mice for in vivo drug treatment. The detailed protocol was described as before [17], [18]. Briefly, 21 mice aged 4 to 6 weeks were pretreated intraperitoneally with anti-mouse CD122 monoclonal antibody (TM-β1, Rat IgG2b, Bio X cell, USA). After conditioning, each mouse was inoculated via tail vein with 1.0×10^7^ thawed leukemia cells that were suspended in 300 µl PBS. After inoculation, engraftment was monitored by weekly blood collections and FACS analysis. When human CD45^+^ (hCD45^+^) cell proportion in peripheral blood reached 1%, 21 mice were randomly grouped into treatment (vincristine or dexamethasone) and control groups (seven mice for each group). The treatment groups received vincristine (0.5 mg/kg every 7 days for 4 weeks), or dexamethasone (20 mg/kg Monday-Friday for 4 weeks) through intraperitoneal administration [17], [19]. The control group received equal volume of PBS. Since treatment, the percentage of human CD45^+^ cells in the peripheral blood was monitored weekly. The end point was defined as the first indication of morbidity (>20% weight loss, lethargy and ruffled fur). The survival was calculated from the initiation of inoculation until the end point. To confirm the engraftment of leukemia cells in NOD/SCID mice at the end point, immunophenotypical and genetic characteristics were inspected by flow cytometry, immunohistochemistry and RT-PCR. The procedures were described as before [17], [18].

### Assessment of Serum Biochemical and Coagulation Parameters in Murine Models

The leukemia cells from the secondary passage of one *SIL-TAL1^+^* and two *SIL-TAL1^−^* T-ALL models (*SIL-TAL1^−^* a and b) were inoculated to NOD/SCID mice as described above. When hCD45^+^ cell proportion in peripheral blood reached 1%, the mice were randomly grouped into treatment (vincristine or dexamethasone) and PBS control groups (six mice for each group), and then received drug treatment as described above. At the end point, blood serum of the mice was collected for routine serum biochemical and coagulation test. Serum samples from six healthy NOD/SCID mice were also tested to provide normal baseline.

### Statistical Analysis

The analysis of categorical variables was performed using Fisher’s exact test for 2×2 tables. Student’s t test was applied to continuous variables. All the survival data were compared using log-rank test, and graphically represent by Kaplan and Meier analysis. All calculations were performed using the SPSS software version 16.0 (SPSS, Chicago, IL).

## Results

### Clinical Characteristics of the Patients

The incidence of *SIL-TAL1^+^* T-ALL in our study was 24.2% (15/62). The clinical characteristics were compared between *SIL-TAL1^+^* and *SIL-TAL1^−^* patients. As shown in Table 1, the *SIL-TAL1^+^* patients were younger in age (median 17 years, *P* = 0.046), and had higher white blood cell (WBC) count (median 184×10^9^/L) than *SIL-TAL1^−^* patients (median 47.2×10^9^/L, *P* = 0.029). There were significantly more cortical T-ALL in *SIL-TAL1^+^* patients (*P* = 0.008). Extramedullary involvement was common in both *SIL-TAL1^+^* and *SIL-TAL1^−^* patient groups, and no difference was found between them. Interestingly, although there was no difference in the lactate dehydrogenase (LDH) level, the incidence of TLS (9/15, 60%) and DIC (11/15, 73.3%) were significantly higher in *SIL-TAL1^+^* patients (both *P*<0.001).

**Table 1:** Comparison between *SIL-TAL1* ^+^ and *SIL-TAL1^−^* T-ALL patients.

Characteristic	*SIL-TAL1* ^+^ (N = 15)	*SIL-TAL1^−^* (N = 47)	*P*
Median age, (Range) (years)	17 (4–48)	21 (6–61)	0.046
Gender, no. (%)			
Male	14 (93.3)	35 (74.5)	0.118
Female	1 (6.7)	12 (25.5)	
Median WBC, (Range) (×10^9^/L)	184 (21–559)	47.2 (0.77–597)	0.029
Median LDH, (Range) (U/L)	2036 (442–7942)	760 (84–15297)	0.199
Immunophenotype, no. (%)			
Pro-T	0	3 (6.4)	0.028
Pre-T	1 (6.7)	17 (36.2)	
Cortical-T	9 (60)	11 (23.4)	
Mature-T	5 (33.3)	16 (34.0)	
Extramedullary involvement, no. (%)			
Splenomegaly	14 (93.3)	33 (70.2)	0.069
Hepatomegaly	13 (86.7)	29 (61.7)	0.072
Mediastinal mass*	5/9 (55.6)	21/39 (53.8)	0.926
CNS infiltration*	4/12 (33.3)	9/33 (27.3)	0.692
TLS, no. (%)	9 (60)	3 (6.4)	<0.001
DIC, no. (%)	11 (73.3)	5 (10.6)	<0.001
Death before induction therapy	4 (26.7)	2 (4.3)	0.011
CR, no. (%)	10/11 (90.9)	41/45(91.1)	0.983

LDH, lactate dehydrogenase; WBC, white blood cell; CNS, central nerves system; TLS, tumor lysis syndrome; DIC, disseminated intravascular coagulation; CR, complete remission.

* Not all patients were evaluated.

### TLS and DIC were Associated with Early Death in SIL-TAL1^+^ Patients

**Figure 1:**
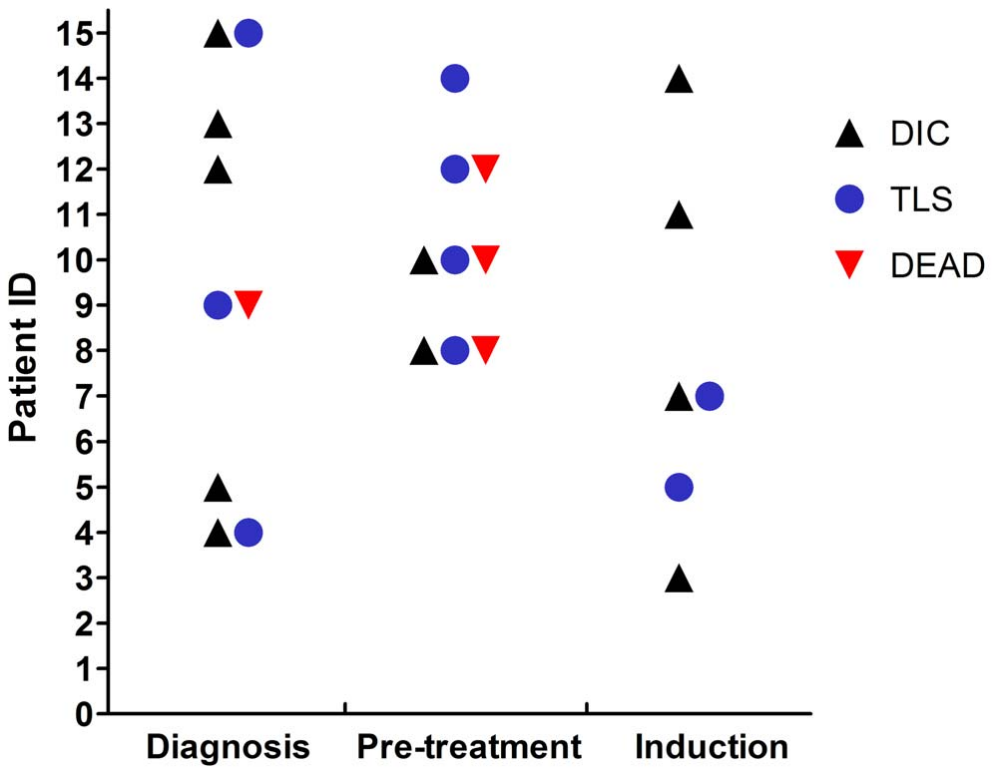
Onset of TLS and DIC. TLS and DIC can occur at any stage, with relatively later onset of TLS in some cases. Early death was observed in 4 patients. Patient 9 died before treatment; patient 8 and 10 died of hemorrhage during leukapheresis at day 1; patient 12 had overt DIC at diagnosis, but died of hemorrhage 7 days after pre-treatment.

As *SIL-TAL1^+^* patients had a higher incidence of TLS (9/15, 60%) and DIC (11/15, 73.3%) (Table 1), we evaluated each of the 15 *SIL-TAL1^+^* patients for DIC and TLS grading in Table S2 according to the diagnostic criteria mentioned before [15], [16]. For the 9 patients developed TLS, 4 patients were classified as grade I and the rest 5 were in grade II. The onset of TLS and DIC was shown in Figure 1. Three patients had TLS at diagnosis; the other 6 patients developed TLS when pre-treatment or induction therapy began. Though we had more DIC cases, the occurrence of DIC was almost parallel with TLS except for the earlier onset in some cases, with 5 occurred at diagnosis, 2 at pre-treatment stage and 4 after induction therapy. Moreover, we noticed a high mortality (4/15, 26.7%) in *SIL-TAL1^+^* patients before induction therapy. All of the 4 patients had TLS, while 3 of them had DIC and died of severe hemorrhage. On the contrary, only 2 patients died before induction in *SIL-TAL1^−^* group (2/47, 4.3%). The difference was statistically significant (*P* = 0.011).

### Treatment and Outcome of Patients

**Figure 2:**
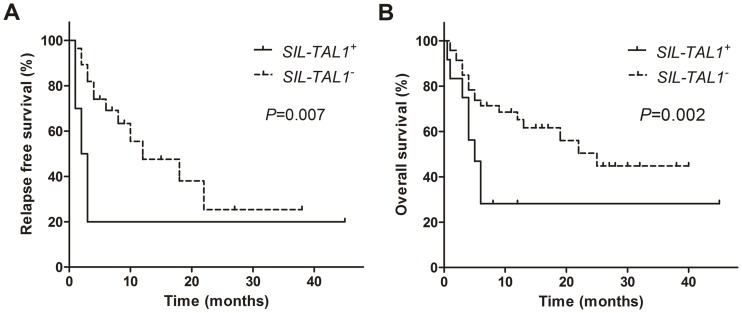
Kaplan–Meier estimates of (A) overall survival (OS), and (B) relapse free survival (RFS). The median OS was 4 months and 25 months for S*IL-TAL1^+^* and S*IL-TAL1^−^* patients (*P* = 0.002), respectively; the median RFS was 2 months and 12 months for S*IL-TAL1^+^* and S*IL-TAL1^−^* patients (*P* = 0.007), respectively.

After pre-treatment and induction therapy, most patients had well responses. The complete remission (CR) rate was 90.9% (10/11) for the *SIL-TAL1^+^* patients and was 91.1% (41/45) for *SIL-TAL1^−^* patients (*P* = 0.983). However, all the *SIL-TAL1^+^*patients relapsed in a period no longer than 4 months. The median RFS was only 2 months (Figure 2A). The overall survival was also poor. Four patients died within 1 month after relapse. The median OS of *SIL-TAL1^+^* patients was 4 months (Figure 2B). In comparison, the *SIL-TAL1^−^* patients had significantly longer RFS (Figure 2A, median 12 month, *P* = 0.007) and OS (Figure 2B, median 25 month, *P* = 0.002) than *SIL-TAL1^+^* patients.

### Disease Phenotype and Outcome of the *SIL-TAL1^+^* Murine Models

**Figure 3:**
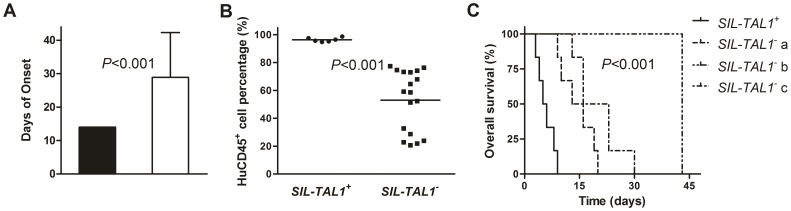
Comparison between murine models inoculated with cells from *SIL-TAL1^+^* and *SIL-TAL1^−^* T-ALL patients. (A) The time of disease onset was 14±0 day for S*IL-TAL1^+^* models and 28.9±13.4 days for S*IL-TAL1^−^* models (*P*<0.001). (B) The percentage of human CD45^+^ cells in mice peripheral blood was 96.3±1.5% for S*IL-TAL1^+^* models and 53.0±21.8% for S*IL-TAL1^−^* models (*P*<0.001). (C) The median survival for S*IL-TAL1^+^* models was 5 days, which was shorter than any of the three *SIL-TAL1^−^* models: a (median 13 days, *P* = 0.001), b (median 43 days, *P* = 0.001), and c (median 16 days, *P* = 0.001). There were significant differences between the four groups (*P*<0.001).

The mice transplanted with *SIL-TAL1^+^* T-ALL cells (*SIL-TAL1^+^* mice) had earlier disease onset (14±0 days) than those mice transplanted with *SIL-TAL1^−^* T-ALL cells (*SIL-TAL1^−^* mice) (28.9±13.4 days, Figure 3A, *P*<0.001). At the end point, engraftment and infiltration of leukemia cells was detected in multiple organs, which mimicked the clinical characteristics of *SIL-TAL1^+^* T-ALL (Figure S1). The percentage of hCD45^+^ cells in peripheral blood were significantly higher in *SIL-TAL1^+^* mice than those in *SIL-TAL^−^* mice (96.3±1.5% *vs.* 53.0±21.8%, Figure 3B, *P*<0.001). As the OS of each model was compared, we found that the 6 *SIL-TAL1^+^* mice have significant shorter survival than the other 18 *SIL-TAL1^−^* mice (Figure 3C, *P*<0.001).

### Drug Treatment in *SIL-TAL1^+^* Murine Model

**Figure 4:**
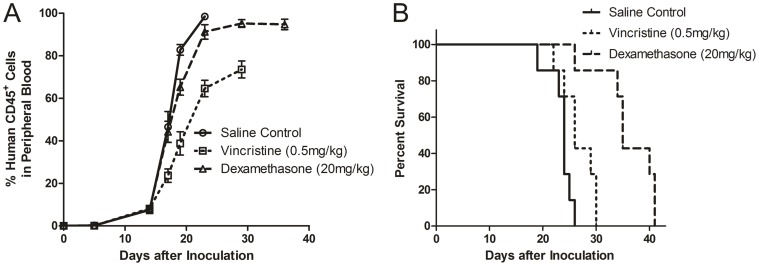
Drug treatment on murine xanotransplantation model. (A) The human CD45^+^ cells were detectable 2 weeks after inoculation in all 3 groups, but the peak of human CD45^+^ cell load was lower in vincristine treated group. (B) The survival of dexamethasone treated group (median 35 days) was longer than the vincristine treated group (median 26 days, *P* = 0.002) and saline control group (median 24 days, *P*<0.001). The difference between saline control and vincristine treatment group was also statistically significant (*P* = 0.033).

We used tertiary passage of mice for drug treatment. As shown in Figure 4A, for vincristine group, the percentage of hCD45^+^ cells could be controlled at no more than 80%, while dexamethasone hardly had effect on suppressing tumor growth. We then compared the survival of mice receiving different treatment strategies. Interestingly, the survival of dexamethasone group (median 35 days) was longer than that of vincristine treated group (median 26 day, *P* = 0.002, Figure 4B), even though the dexamethasone group had higher hCD45^+^ cell level. In the vincristine group, instead, when the hCD45^+^ cell reached near 80%, all mice died soon after vincristine administration. As a result, the vincristine group had slightly longer survival than saline control group (24 days, *P* = 0.033, Figure 4B).

### *SIL-TAL1^+^* Murine Model Had more Evidence of TLS and DIC

Since we found that *SIL-TAL1^+^* patients were associated with TLS and DIC, as well as the paradoxical survival of drug treated *SIL-TAL1^+^* murine models, we carried out the serum biochemical and coagulation test on the drug treated murine models for further confirmation. As shown in table 2 (calculated from Table S3), compared to the *SIL-TAL1^−^* murine models, the *SIL-TAL1^+^* murine models had significantly higher LDH (p = 0.009), potassium (p<0.001) and uric acid levels (p<0.001), which revealed that the *SIL-TAL1* ^+^ models tended to develop TLS. Similarly for the coagulation tests, the *SIL-TAL1^+^* murine models had significantly prolonged prothrombin time (PT, p<0.001) and activated partial thromboplastin time (APTT, p<0.001), lower fibrinogen level (p<0.001) and marginal elevated D-dimer level (p = 0.076), indicating a disregulated coagulation status in these mice. Besides, in the *SIL-TAL1^+^* murine models, the vincristine treatment group had longer PT and APTT, and higher D-dimer and fibrinogen degradation product (FDP) level than the dexamethasone treatment group (all p<0.001), demonstrating that vincristine treatment was more likely to induce DIC.

**Table 2:** Comparison of serum biochemical and coagulation parameters between *SIL-TAL1* ^+^ and *SIL-TAL1^−^* murine models.

Parameters	*SIL-TAL1* ^+^ (N = 18)	*SIL-TAL1^−^* (N = 36)	*P*	*SIL-TAL1* ^+^		*P*
				VCR (N = 6)	DEX (N = 6)	
**Median LDH**,(Range) (U/L)	9122(3038–15223)	6417(2707–8487)	0.009	12720(11763–15223)	3468(3038–4375)	<0.001
**Median potassium**,(Range) (mmol/L)	9.69(8.77–11.35)	7.35(6.21–8.43)	<0.001	9.50(8.77–10.63)	9.65(8.93–10.66)	0.735
**Median calcium**,(Range) (mmol/L)	2.57(2.31–2.77)	2.59(2.36–2.73)	0.662	2.53(2.34–2.72)	2.66(2.48–2.77)	0.158
**Median phosphate**,(Range) (mmol/L)	2.47(2.27–2.94)	2.71(2.44–3.07)	0.005	2.42(2.29–2.51)	2.37(2.27–2.53)	0.628
**Median uric acid**,(Range) (µmol/L)	280(256–318)	181(126–218)	<0.001	270(256–285)	273(256–286)	0.720
**Median PT**,(Range) (second)	18.9(12.5–24.6)	13.6(11.6–14.6)	<0.001	21.9(19.5–24.6)	13.9(12.5–15.3)	<0.001
**Median APTT**,(Range) (second)	64.7(41.8–88.4)	31.2(26.6–37.5)	<0.001	83.9(75.4–88.4)	45.3(41.8–47.0)	<0.001
**Median fibrinogen**,(Range) (g/L)	0.68(0.52–0.77)	1.62(1.25–2.26)	<0.001	0.65(0.52–0.72)	0.70(0.58–0.77)	0.200
**Median D-dimer**,(Range) (mg/L)	0.24(0.16–0.35)	0.22(0.16–0.32)	0.076	0.30(0.26–0.35)	0.20(0.16–0.24)	<0.001
**Median FDP**,(Range) (mg/L )	1.09(0.42–1.89)	1.06(0.33–1.31)	0.163	1.74(1.63–1.89)	1.09(0.89–1.32)	<0.001

LDH, lactate dehydrogenase; PT, prothrombin time; APTT, activated partial thromboplastin time; FDP, fibrinogen degradation products; VCR, vincristine; DEX, dexamethasone.

## Discussion

*SIL-TAL1* rearrangement is a common form among heterogeneous *TAL1* related alterations. Only a few clinical researches have been hitherto performed to describe the features of *SIL-TAL1^+^* T-ALL. In the present study, we analyzed the characteristics of *SIL-TAL1^+^* T-ALL retrospectively in 15 Chinese patients presented to our department, and made a comparison with the other 47 *SIL-TAL1^−^* T-ALL patients administered during the same period. The incidence of *SIL-TAL1^+^* T-ALL in our study was comparable with previous literatures [4]–[8], [10]–[12]. We showed that the *SIL-TAL1^+^* patients were linked with relatively younger age, higher WBC count, more cortical immunophenotype, and a poor outcome. These clinical features were similar to the Brazilian report [6]. However, we noticed that a large proportion of *de novo* patients with *SIL-TAL1* developed TLS and DIC during, or even before chemotherapy, and resulted in a higher mortality in the early stage, which has also been simulated by our *SIL-TAL1* ^+^ murine model. These features have not yet been observed in any form of *TAL1* related alterations. Higher tumor burden might account for these features, which was indicated by extremely higher WBC count and elevated LDH level than Brazilian reported in the peripheral blood.

Hyperleukocytic leukemia is conventionally defined as leukemia with an initial WBC count or blast count greater than 100×10^9^/L and associated with a very high early mortality rate (20∼40%) [20]. Symptoms of hyperleukocytosis are primarily due to leukostasis and subsequent endothelial damage [20]. The clinical implication of hyperleukocytosis of ALL is different from those in acute myeloid leukemia (AML) [21], [22]. Eguiguren et al. reported that in pediatric ALL, hyperleukocytosis is significantly associated with age of less than 1 year, T-cell lineage, leukemic cell ploidy <50 chromosomes, presence of a mediastinal mass, and central nervous system leukemia at diagnosis [23], some of which were also presented in our study. Despite a higher WBC count in ALL than AML, leukostasis is rarely seen in ALL (2% to 6%), and management focuses primarily on the treatment of TLS and DIC [22].

TLS is an emergency of malignant tumors caused by rapid degradation of tumor cells. WBC count over 100×10^9^/L and LDH over 2 times of upper limit of normal level are considered of high risk to develop TLS [24]. Based on the risk stratification criteria, most of our patients with *SIL-TAL1* belonged to the high risk group, thus we were not astonished to find that two thirds of these patients developed TLS. Usually TLS is treatment related, while in some cases with high tumor load, TLS may occur spontaneously [24]. We did observe three patients with *SIL-TAL1* developed TLS before chemotherapy, but most TLS were controllable, since all the TLS cases were within grade I or II, which were not life-threatening.

Coagulopathy was another characteristic manifestation in our study. We discovered 11 patients with *SIL-TAL1* developing overt DIC in different degrees according to the scoring system given by International Society on Thrombosis and Hemostasis (ISTH). In previous literatures, the DIC was found in about 10% of adult ALL patients, and less than 5% in childhood ALL [25], [26]. In our study, the incidence of DIC was much higher in patients carrying *SIL-TAL1*, and most DIC accompanying with TLS occurred in the early stage of induction therapy, which indicated that DIC may also be related to the breaking down of leukemia cells during chemotherapy. Meanwhile, three *SIL-TAL1^+^* patients died from severe hemorrhage, prompting DIC as a more lethal event than TLS. Among the three patients, one had DIC at diagnosis, the other two patients developed DIC during or soon after leukapheresis and died within one day, in whom coagulopathy might be accelerated by the mechanical destruction from leukapheresis.

*SIL-TAL1^+^* ALL had a poor outcome, which was indicated by the high relapse rate and the short RFS and OS of our patients. The treatment outcome of relapsed ALL is highly heterogeneous. From several large cohort studies in children or adults, the prognosis of recurrent ALL was poor [27], [28]. In adults, relapsed patients with young age and long duration of the first remission were predicted to have a good outcome after salvage therapy; otherwise, whatever the prior treatment, relapsed patients cannot be rescued using currently available regimens [28]. Our data showed that most of the *SIL-TAL1^+^* patients cannot survive for 1 month after relapse. These observations suggested that although treatment outcome in patients with T-ALL has improved in recent years, patients with relapsed disease continue to have a dismal outcome and the prognosis of *SIL-TAL1^+^* ALL was probably poor.

With respect to the murine models, our *SIL-TAL1^+^* xenograft model, derived from a seven years old boy, also exhibited rapid increase of blast cells in peripheral blood and overt multiple organ infiltration, as well as a poor survival, suggesting that *SIL-TAL1^+^* T-ALL was highly proliferative and aggressive. Glucocorticoids have long been used in the treatment of ALL and glucocorticoid resistance is an adverse prognostic factor in childhood ALL [29], [30]. In our in vivo drug treatment, dexamethasone could hardly retard the progression of engraftment. Given the data from ALL-BFM 95 trial, which indicated that the prognosis was poor in childhood T-ALL with prednisone resistance [31], this boy may be linked to a dismal outcome. Another finding was that the survival of our murine model was not improved by vincristine, although it could suppress tumor growth. Death of mice soon after vincristine administration was similar to the clinical occurrence of DIC, and this presumption was further confirmed by the coagulation test on the mice. The paradox that dexamethasone group had longer survival may be resulted from the protective effect of dexamethasone on endothelial cells against leukostasis.

On the basis of our clinical observations and murine models, our study revealed that *SIL-TAL1^+^* ALL represented a distinct ALL subtype, which was characterized by a higher WBC count and a predominant cortical T-ALL immunophenotype. Moreover, *SIL-TAL1^+^* ALL tended to develop TLS and DIC and related with inferior outcome. These findings could aid the individual therapeutic stratification for T-ALL patients and attract more attention for the treatment of original disease. Besides, our *SIL-TAL1* ^+^ xenotransplantation model could be used as model of TLS and DIC for drug assessment. However, the present study was retrospective and based on a relatively small number of patients from single clinical center and a few murine models. Therefore, expanded multicenter clinical trial and experimental.

## Supporting Information

Figure S1 Engraftment of leukemia cells in *SIL-TAL1* ^+^ xenograft model. (A) All nucleated cells were gated by light scattering properties. (B–E) Monitoring the engraftment of human leukemia cells by flow cytometry. Cells were stained with FITC-conjugated anti-human CD45 antibody resulting in the percentage of human cells (hCD45^+^, %) as shown in B–E. Leukemia cells in peripheral blood (PB) at 2 weeks post inoculation (B) and at the end point (C). Leukemia cells in bone marrow (BM, D) and spleen (SP, E) at the end point. (F) The spleen from *SIL/TAL1* ^+^ xenograft model (right) was larger than the normal one (left). (G–K) Immunohistochemistry staining of the leukemia cells on sections from liver (G), spleen (H), lung (I), kidney (J) and heart (K) of a murine model at the endpoint. (L) FISH analysis of nucleated cells in the peripheral blood from a murine model. Cells were detected with a fluorescence-labeled probe and counterstained with DAPI. The probe (p17h8) was human specific. Only engrafted human cells would present two green signals, whereas the cells from mouse would not. (M) RT-PCR analysis of the SIL-TAL1 fusion transcript from peripheral blood of the murine model at the endpoint. The internal positive control e2a (690 bp) and amplifed product (∼371 bp) are shown in lanes 1 and 2, respectively.
(TIF)

Table S1 Engraftments of leukemia cells in NOD/SCID mice.
(DOC)

Table S2 Treatment regimens and outcome of *SIL-TAL1^+^* patients.
(DOC)

Table S3 The original data of the serum biochemical and coagulation tests on the mice models.
(XLS)
