# Supplementary figures and images for: SIL-TAL1 Rearrangement is Related with Poor Outcome: A Study from a Chinese Institution
Source: PLoS One. 2013 Sep 9;8(9):e73865. doi: 10.1371/journal.pone.0073865 (PMC3767609; doi:10.1371/journal.pone.0073865)

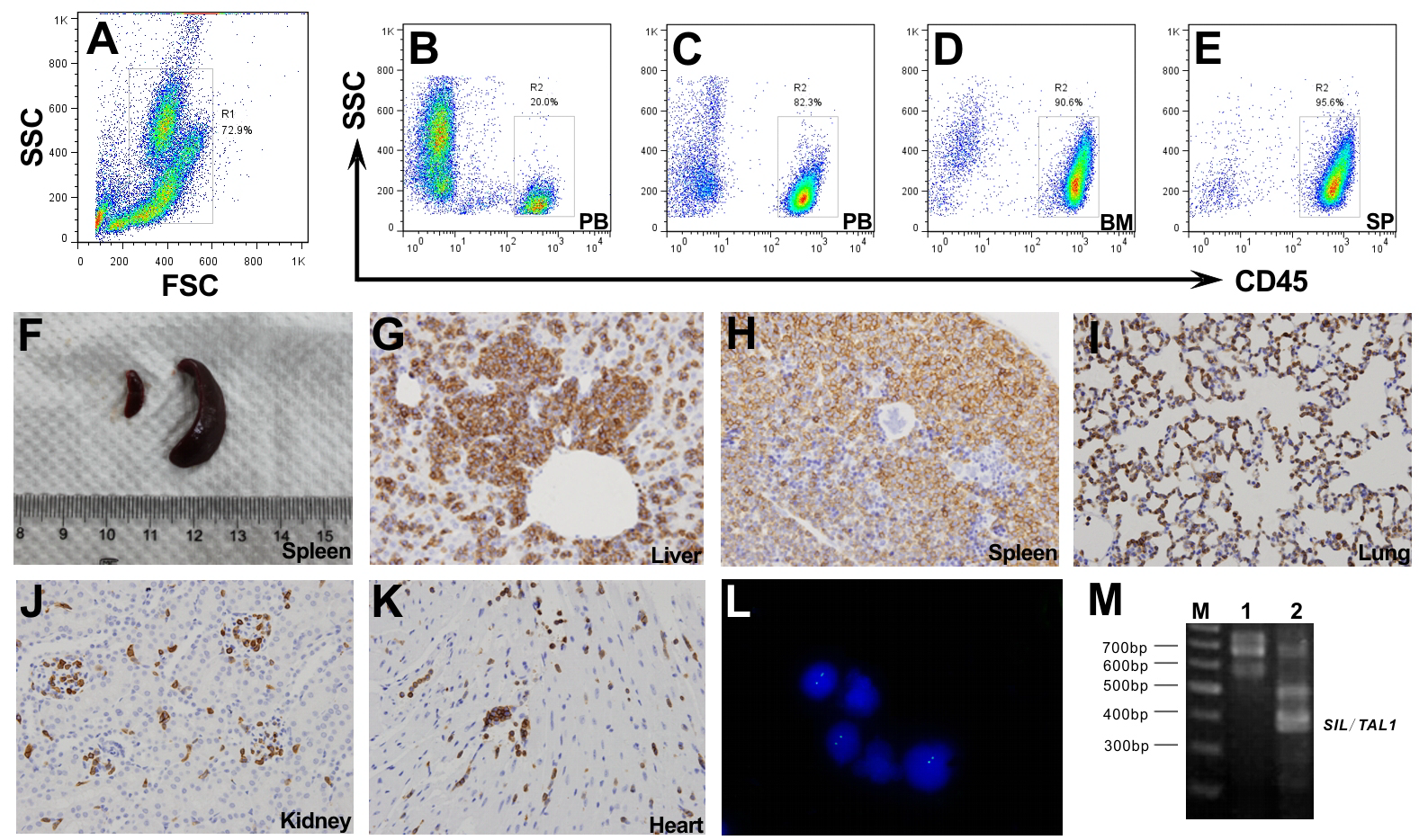

Supplement: Figure S1 — Engraftment of leukemia cells in SIL-TAL1 + xenograft model. (A) All nucleated cells were gated by light scattering properties. (B–E) Monitoring the engraftment of human leukemia cells by flow cytometry. Cells were stained with FITC-conjugated anti-human CD45 antibody resulting in the percentage of human cells (hCD45+, %) as shown in B–E. Leukemia cells in peripheral blood (PB) at 2 weeks post inoculation (B) and at the end point (C). Leukemia cells in bone marrow (BM, D) and spleen (SP, E) at the end point. (F) The spleen from SIL/TAL1 + xenograft model (right) was larger than the normal one (left). (G–K) Immunohistochemistry staining of the leukemia cells on sections from liver (G), spleen (H), lung (I), kidney (J) and heart (K) of a murine model at the endpoint. (L) FISH analysis of nucleated cells in the peripheral blood from a murine model. Cells were detected with a fluorescence-labeled probe and counterstained with DAPI. The probe (p17h8) was human specific. Only engrafted human cells would present two green signals, whereas the cells from mouse would not. (M) RT-PCR analysis of the SIL-TAL1 fusion transcript from peripheral blood of the murine model at the endpoint. The internal positive control e2a (690 bp) and amplifed product (∼371 bp) are shown in lanes 1 and 2, respectively. (TIF) [file pone.0073865.s001.tif]
