# Supplementary material for: SIL-TAL1 Rearrangement is Related with Poor Outcome: A Study from a Chinese Institution
Source: PLoS One. 2013 Sep 9;8(9):e73865. doi: 10.1371/journal.pone.0073865 (PMC3767609; doi:10.1371/journal.pone.0073865)
Supplement: Table S1 — Engraftments of leukemia cells in NOD/SCID mice. (DOC) [file pone.0073865.s002.doc]

**Table S1. Engraftments of leukemia cells in NOD/SCID mice.**

| **Patient** | **Cells inoculated (×10^6^)** | **Mice engrafted** | **Initial engraftment (Days±SD)** | **Overall survival (Days±SD)** | **Final Engraftment Level (hCD45^+^%, Mean±SD)** |
| --- | --- | --- | --- | --- | --- |
| *SIL/TAL1*^-^ a | 10 | 6 | 14±0 | 14.5±4.6 | 61.6±10.1 |
| *SIL/TAL1*^-^ b | 10 | 6 | 28±0 | 43±0 | 72.2±5.0 |
| *SIL/TAL1*^-^ c | 10 | 6 | 44.8±6.3 | 20.2±6.3 | 25.1±4.7 |
| *SIL/TAL1*^+^ | 10 | 6 | 14±0 | 5.8±2.3 | 96.3±1.5 |

SD, standard deviation
