# Supplementary material for: SIL-TAL1 Rearrangement is Related with Poor Outcome: A Study from a Chinese Institution
Source: PLoS One. 2013 Sep 9;8(9):e73865. doi: 10.1371/journal.pone.0073865 (PMC3767609; doi:10.1371/journal.pone.0073865)
Supplement: Table S2 — Treatment regimens and outcome of SIL-TAL1+ patients. (DOC) [file pone.0073865.s003.doc]

| ID | Pre-treatment | Induction regimen | TLS Grading | DIC Scoring | CR | Relapse |
| --- | --- | --- | --- | --- | --- | --- |
| 1 | None | CHOP | - | Non-overt | No | - |
| 2 | Dex + CTX | DVCP | - | Non-overt | Yes | Yes |
| 3 | Dex + CTX | VDLP | - | Overt | Yes | No |
| 4 | Dex + CTX | VDLP | II | Overt | Yes | Yes |
| 5 | Dex + CTX | VDLP | I | Overt | Yes | Yes |
| 6 | Dex + CTX | DVCP | - | Non-overt | Yes | Yes |
| 7 | None | hyper-CVAD | II | Overt | Yes | Yes |
| 8 | leukapheresis | None | II | Overt | - | - |
| 9 | Dex + CTX | None | II | Non-overt | - | - |
| 10 | leukapheresis | None | I | Overt | - | - |
| 11 | Dex + CTX | VDLP | - | Overt | Yes | Yes |
| 12 | Dex + CTX | None | I | Overt | - | - |
| 13 | None | DVCP | - | Overt | Yes | Yes |
| 14 | Dex + CTX | VDLP | I | Overt | Yes | No |
| 15 | Dex + CTX | DVCP | II | Overt | Yes | Yes |

**Table S2. Treatment regimens and outcome of *SIL-TAL1^+^* patients.**

TLS tumor lysis syndrome; DIC disseminated intravascular coagulation; CR complete remission; Dex dexamethasone; CTX cyclophosphamide; CHOP cyclophosphamide, doxorubicin, vincristine and prednisone; DVCP doxorubicin, vincristine, cyclophosphamide and prednisone; VDLP vincristine, doxorubicin, L-asparaginase and prednisone; hyper-CVAD cyclophosphamide, vincristine, anthracycline and dexamethasone.
